# Does the Connectivity of Urban Public Green Space Promote Its Use? An Empirical Study of Wuhan

**DOI:** 10.3390/ijerph17010297

**Published:** 2020-01-01

**Authors:** Yuping Dong, Helin Liu, Tianming Zheng

**Affiliations:** 1 School of Architecture and Urban Planning, Huazhong University of Science and Technology, Wuhan 430074, China; youkie@hust.edu.cn (Y.D.); tm1996@hust.edu.cn (T.Z.); 2 Centre for Urban and Rural Planning Support Research, Huazhong University of Science and Technology, Wuhan 430074, China

**Keywords:** pubic green space use, connectivity of pubic green space, Location Based Service data, impact factors, Wuhan

## Abstract

A high greenness level can enhance green space use and outdoor physical activity. However, rapid urbanization and high-density development have led to the loss or fragmentation of green space, especially urban public green space (PGS). With the aim of increasing the health benefits from PGS, some planners and researchers suggest connecting existing PGSs to encourage urban residents to use the PGS, and thus, to improve public health. Does this suggestion stand with robustness? By taking 42 sub-districts in the inner area of Wuhan as the study objects, this paper examines the correlation between the connectivity of PGS and its use. We also explore how the characteristics of PGS and the facilities/functions in the neighboring areas influence this relationship by using Location Based Service data (WeChat-Yichuxing data), point of interest (POI) data, and remote-sensing image, etc. Using Regression Analysis, we found that there is no high correlation between PGS use and its connectivity. The possible causes might be attributed to the fact that PGS use is profoundly influenced by multifaceted competing impact factors, and no one can stand dominantly. It is interesting to see that the density of companies is positively, but slightly, related to PGS use.

## 1. Introduction

Green space, as an important part of urban environment, has been found to produce various health-related benefits, like lowering the odds of being overweight or obese [1], reducing psychological distress [2], promoting general wellbeing [3], improving self-esteem and mood [4], relieving stress and depression [5], etc. Green space, especially public green space (PGS), is considered as a significant catalyst in encouraging physical activity, as it could be conducive to the improvement of frequency, intensity, and duration of outdoor physical activity [1,6,7,8,9,10]. Taking physical activity within such green environments could produce more benefits than when in non-green settings [11,12,13]. Hence, a variety of strategies and plans stress the importance of green space in improving public health and encourage residents to increase green space use [14,15].

However, rapid urbanization and land-use densification lead to the constant decrease and fragmentation of open space, especially green space in urban areas [16,17,18]. This means that green space use and physical activity generated by green space might be suppressed by such built environment changes. On the one hand, continually shrinking urban green space cannot meet residents’ growing demands, due to the inadequate and scarce provision; on the other hand, the unevenness of urban green space distribution potentially could result in unequal accessibility and availability for residents [7,19,20,21]. Nevertheless, in the intensively compact inner urban area, it is almost impossible to widely build large new green spaces or largely change the structure of existing urban green space systems to promote PGS use and physical activity [18,22,23]. Considering that, several researchers have argued that enhancing the connectivity of these green spaces by referring to the principals of landscape ecology is likely a relatively economical and efficient way to handle with the aforementioned challenges [22,24,25].

In the urban context, well connected green spaces not only reflect more ecology and sustainability, contributing to improved residents’ perception of well-being; but they also tend to increase the accessibility to green space system [26]. In other words, the connectivity of green space could improve the micro-climate, living environment, urban landscape quality, etc., which, in turn, has the potential to enhance green space use [27]. The connectivity of green space plays a significant role in the movement of organisms and processes, which is beneficial to biodiversity conservation [28]. Evidence has shown that biodiversity is an important factor in influencing green space use and green spaces with higher biodiversity (such as variety in birds and richness in vegetation) are more likely to be visited [29]. In addition, the connectivity of green space is found to have a positive impact on the perception of greenness and safety [26]. Subsequently, it can improve the attractiveness of green space and induce green space use, to a certain degree. Lastly, well connected green spaces could form a continuous green space system, which encourages runners and cyclists to use the green space [30,31].

Although both the connectivity and accessibility of green space can reflect the ease of access to green space, they are still two different concepts. From the perspective of structure, accessibility tends to stress the distance (Euclidean distance/road network distance/self-reported distance) or time it takes to travel from the starting point to the point of green space (such as entrance, exit, and centroid). The focus is on the linear spatial relationship between two points. However, the calculation of the connectivity is based on nodes and links. The connectivity sheds light on the network of the topological structure among habitat patches or green spaces. In view of the analysis objectives, accessibility studies are on the convenience from certain places to individual green spaces. According to the relevant research, most of these places are non-green environments, like homes, schools, workplaces, etc. In contrast, connectivity takes all green spaces (within a certain research scope or within a certain defined distance) into consideration and emphasizes the access between them. Accessibility tends to regard green space as the destinations and stresses the importance of independent individual green spaces [32], while connectivity places more emphasis on the network of all individual green spaces within a certain area, and each green space is treated as a node with different significances across the whole network [33,34]. Therefore, connectivity could partly reflect accessibility between two arbitrary nodes (green spaces) within a certain scope, but not be necessarily equal to accessibility. Furthermore, the accessibility of green space is the essential precondition for the influence of connectivity on green space use, because residents need to access to the green space system first, then it is possible that good connection among green spaces would lead to users’ (such as the commuters, runners and cyclists) continuous use of the green space system, while bad connectivity might shorten the time people spend in the green space system.

Connectivity was first put forward to measure the movement of wildlife among dispersed habitat patches in ecology theory [27]. A series of studies have explored the impact factors of connectivity [35], ecological benefits of connectivity [36], construction and optimization of ecological networks [37], measurement and evaluation of connectivity [38], and structural assessment of green spaces’ connectivity [27]. Most of these focus on how the connectivity of habitat patches or green spaces influence their use by the wildlife and its ecological effects on human beings. However, apart from the ecology-related benefits, few studies have considered how connectivity may interact with human beings. In other words, research on the influence of the connectivity of green space upon its use by human beings is limited. Hence, we refer to such concept of ecology theory to analyze if the connectivity of green space is related with its use (namely, green space use), by regarding human beings as the target species, and if other relevant factors have a significant impact on this relationship.

According to previous studies, there are various indicators used to reflect green space use (by human beings), such as the frequency of visiting green space, the duration of exposure to green space, and the types and intensity of physical activity within green space. Most of them are calculated based on the individual-level data by self-report, which might cause the ‘Hawthorne Effect’ [39], and cannot evaluate the actual green space use from a relatively holistic perspective [40,41]. However, with the development and wide application of technologies of location-based services (LBS), such as the global system for mobile communication (GSM), the global positioning system (GPS), the social networking services (SNS) and wireless broadband hotspot, the acquisition of large-scale and high-quality individual-based spatiotemporal data becomes possible, and these ubiquitous individually-volunteered data provide a new method to objectively describe and understand certain spatial structures [42,43]. 

LBS data is real-time data based on geo-location. Firstly, it can collect individual’s real locations, which include multiple dimensions of time and space [44]. Secondly, it has higher quality and accuracy than the data obtained by traditional means [45]. Thirdly, real-time movement information on individuals can be obtained based on the location-based service, which thereby supports the analysis of individual’s spatiotemporal behavior trajectories [43]. Lastly, much higher capabilities of mobile computing and communication, as well as analysis, can reduce information loss in the process of coding behavioral data and allow for large-scale spatiotemporal data analysis [45]. Currently, multi-source LBS data that has been mined and used in city-related research includes mobile signaling data, GPS data, bus smart card data (SCD), Tencent data, and Baidu index data, etc. [46]. Scholars have applied them to different urban problems, such as urban vitality [47], green space use [48], the relationship between occupation and residence [42], and transportation models [45], on the basis of their temporal and spatial characteristics. 

Compared with traditional data, LBS data can mirror larger, more representative samples, as well as more refined phenomena and results in fine spatiotemporal granularity. Considering that, this paper applies the average daily population density within the studied green space to mirror its physical use on the basis of Location-Based Service (LBS) data. Nevertheless, as Muhammad et al. (2019) comment that such data is more “a supplement to than a substitute of traditional data sources, while taking a decision on policy-making associated with urban planning and city functionalities” [49], this kind of data lacks of the attributes that traditional data has, such as demographic characteristics, socioeconomic characteristics, etc. In this sense, the PGS use represented by the population density in this paper can only reflect use quantity (at the population level), but no other socioeconomic features.

Overall, the aim of this paper is to explore if the connectivity of green space positively correlates with its use, and if other relevant factors mediate or moderate this relationship by using LBS data. As public green spaces (PGSs) are accessible green infrastructures and free resources to all residents; in this study, we only take PGSs, but not all the green spaces into consideration. With this goal, this paper is organized as follows: Section 2 introduces the study area, as well as the collection, calculation, and analysis methods of the concerned data/indicators; Section 3 and Section 4 present the results of this study and discusses the implications, as well as the underlying reasons from the perspective of urban planning; Section 5 summarizes the conclusions and puts forward potential issues for further research.

## 2. Materials and Methods 

### 2.1. Research Framework

This study aims to shed light on the influence of the connectivity of PGS on urban residents’ use of it (namely PGS use), and explore what other relevant factors would play in this interaction. That is, the central idea is to check if well connected PGS could promote its use. Generally, if a positive correlation between them is identified, then we can conclude that higher connectivity would have the potential to promote higher PGS use. However, this relationship may be established not only via their direct interaction, but also through other paths where other relevant factors play key roles. Regarding this, this study goes a step further to see if there are any other impact factors that moderate or mediate PGS use.

**Figure 1:**
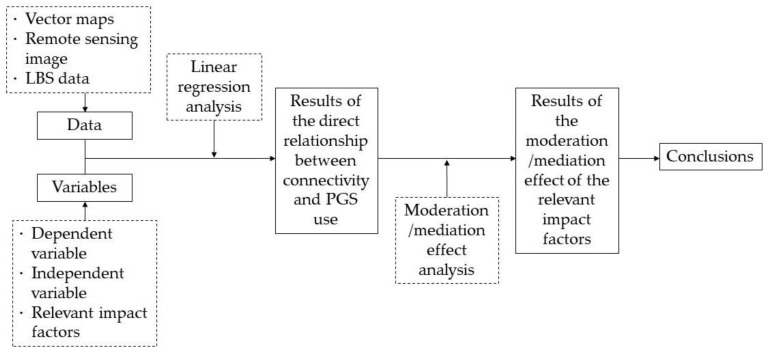
Research framework.

With the research goal, as depicted by Figure 1, the first step is to conduct the general linear regression analysis. The requisite data are the vector map of the study area (Wuhan), the corresponding remote sensing image and the LBS data. The dependent variable of PGS use is represented by the average daily population density mined through the LBS data from Yichuxing; and the independent variable of connectivity among PGSs are calculated by referring to the vector map of the green space of Wuhan. Through this process, it is possible to generate results of the direct relationship between connectivity and PGS use. Then a moderation/mediation effect analysis is executed to check how the relevant impact factors influence the direct relationship between PGS use and connectivity. It is worth noting that there may be different moderation and mediation modes which entail more than one moderation/mediation effect analysis in this process. By integration of these results, then it is possible to draw the conclusions that would answer the question as stated by the title of this paper.

### 2.2. Study Area

Wuhan, the capital city of Hubei province and one of China’s megapolises located in middle China, is the transportation hub, the core of the economy, technology, as well as commerce trade logistics in the Triangle of Central China. It is divided by the Yangtze River and Han River into three parts, namely Hankou, Wuchang, and Hanyang. There are 13 districts, and each contains several sub-districts/Jiedaos (sub-district, also translated as Jiedao, the lowest administrative unit in China). The urbanization rate is 80.29%, with approximately 647 million residents living in the urban area in 2018 [50]. It is reported that the frequency of residents taking physical activity in Wuhan is relatively low and usually no more than three days a week [51]. In 2015, only about 5% of the residents regularly participated in physical activity in Wuhan, and most residents lived a sedentary lifestyle [52]. Moreover, compared with the average level of 1.57 m^2^ per capita in China [53], Wuhan has a lower average ground area dedicated to sports. It is 1.43 m^2^ per capita [52]. 

With the socio-economic transformation and public awareness of the heath-related benefits of green space, the desire for an equitable opportunity of acquiring green space in Wuhan is urgent [54]. After analyzing the spatio-temporal disparity between demand and supply of PGS from 2000 to 2014, Xing et al. (2018) found that although the quantity of PGS supply has increased, the population has grown faster [55]. As a result, the supply of PGS does not meet the demand of residents. The places where new PGSs are constructed do not match with the areas where there is a relatively high population density, which further widens the gap between supply and demand. Analysis results show that the percentage of the population within a 5 min walking distances of the PGS entrance is less than 3%, and within 10 min is less than 10%. Most residents need to walk 30-35 min to reach a PGS.

Such conflicts between provision and demands are more prominent in those old town areas in Wuhan [54]. For one thing, the provision of green space is insufficient for the local residents in most of those places, as the overcrowded built-up environment and impermeable land-use are dominant. Compared with new towns, old ones have less vitality, a worse sense of safety, less undeveloped space, and lower construction standards for green space, which are all harmful to the building of social support and social capital. This is because green space use can promote social interaction and contact, decrease crime aggregates, and improve social cohesion [56,57,58].

**Figure 2:**
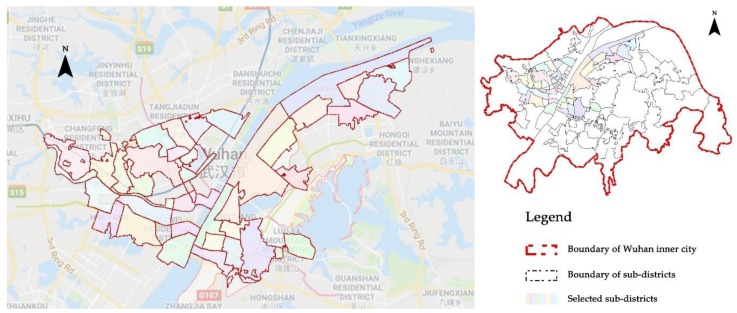
The study area and the 42 sub-districts in Wuhan.

In light of the above facts, it is significant to examine the relationship between the connectivity of PGS and its use in Wuhan, particularly the inner urban area. Considering the situation that the construction and management of most green space, as well as the census is consistent with administrative division, we take the sub-district as the basic analysis unit. On the one hand, it helps to set a standard unit for all the data; on the other hand, it would make the application of conclusions to urban management practice easier. Here we select 42 such units from the old town areas of Wuhan. As depicted in Figure 2, most of them are small in size and clustered alongside the Yangtze River and the Han River. 

### 2.3. Indicator Definition

#### 2.3.1. PGS Use

A series of studies have reported that whether the type of green space use is for utility (like commuting) or leisure (like relaxing), and whether the use time is long or short, the PGS always generates health-related benefits for its users [4]. In other words, as long as the person is present in a PGS, it is a kind of green space use. Accordingly, we assume that once the mobile phone is positioned within PGS, the owner of the phone is regarded as the user of PGS. Hence, we employ the density of population within PGS as the indicator with the help of LBS data to mirror PGS use in this study. Moreover, since the LBS data is obtained from the mobile terminal and presents in the form of longitude and latitude, this means that the criteria for whether the person uses the green space or not are determined by the geographic information without requiring individual self-reporting. Therefore, not only can we avoid subjective errors, but we can objectively demonstrate PGS use by the actual population.

#### 2.3.2. The Connectivity of PGS

There have been many indicators applied to measure the connectivity of ecological networks, including total number of links, size of the largest component, number of components, Harary index, normalized Harary index, integral index of connectivity (IIC) etc.; however, after comparison, IIC is found to be a relatively suitable index to show the connectivity of habitat patches [59]. IIC is an index that can adequately mirror the availability of habitat and identify the most significant habitat patches for maintaining landscape connectivity [33]. It requires the calculation of shortest paths between every pair of nodes, which corresponds with the movement habits of human beings whereby they tend to choose the shortest pathways to get to their destinations. Considering this, IIC is used to measure and reflect the connectivity of PGS in our study. 

#### 2.3.3. Relevant Impact Factors

A large body of studies suggest that green space use is influenced by various impact factors [60]. For example, the characteristics of PGS-greenness, availability, accessibility and quality, etc.-are all found to be able to promote its use [61]. Various facilities and services surrounding PGS are attractive factors for many potential green space users as these elements can be destinations for their multi-level demands [41,62]. Socio-economic-demographic characteristics, such as gender, age, income and education level, also have the potential to moderate or mediate PGS use [61]. Considering that this paper focuses on PGS use at the population level, we choose the relevant impact factors, but no individual information. By referring to the previous studies, we apply a normalized difference vegetation index (NDVI), PGS area per capita, and the ratio of arbor to shrub to measure the characteristics of PGS [63,64]. It worth noting that the indicator of the ratio of arbor to shrub can reflect the aesthetic characteristic of green space quality to a certain degree [65]. Considering the basic needs of local residents, four categories of facility functions around PGS that possibly affect green space use are considered in this study, namely living, working, education and entertainment [41,62]. 

### 2.4. Data Collection

The requisite data is collected by referring to the selected indicators. As shown in Table 1, except for the basic data of sub-districts distribution (vector map with the attribute of actual resident numbers, provided by Wuhan Statistics Bureau), we also need to obtain the map of the public green space, Location-Based Services data, remote sensing image and point of interest data.

#### 2.4.1. Public Green Space

The distribution of public green space within the inner area of Wuhan is extracted from the vector-graph of the green space system provided by the official greening department in Wuhan. Public green space in this study consists of parkland (including a large multi-purpose park, neighborhood park, theme park and pocket park), land for squares (with green space proportion higher than 35%), parts of ecological green space, as well as attached green space that can be freely approached. One hectare or more is proven to be a reasonable area for green space use, especially, for physical activity [66]. Hence, only those green spaces with a size equal to or larger than 1 hectare are counted in in this research.

#### 2.4.2. Location Based Services Data

Location-Based Services (LBS) data in this paper is from Yichuxing, an added-in function of WeChat of Tencent company, and WeChat is the most popular social media app in China. It can reflect population distribution by acquiring users’ mobile terminal real-time position information (longitude and latitude). As long as the users login and give WeChat permission to get their location information, no matter if they use it or not, Yichuxing has acquired their geo-location. In other words, even if WeChat runs in the background, users’ locations can still be positioned by Yichuxing. Thus, the stored value for each statistical unit by Yichuxing is the standardized number of populations using WeChat. Therefore, it can be used to represent the relative population in a certain area. We hired a professional data crawler company to collect this. Data acquisition time in this paper covers a week (seven days), and the weather is non-rainy during this period. We started at 06:00 and ended at 22:00, every day, with two-hour interval between data collections. In other words, there are 63 time points (nine time points/ day * seven days) of such data acquired in total. The contents of such data include ID, longitude, latitude, and v-data, without any information on individual demographic and socioeconomic characteristics. In particular, v-data is the standardized number of populations aforementioned. Here, the points that are positioned within PGS are used to calculate the relative use of public green space.

#### 2.4.3. Remote-Sensing Image

Compared with the data of a traditional survey, the data from Landsat remote sensing is faster, more objective, lower cost, wider range, multi-temporal, and non-destructive [67]. Hence, NDVI derived from it can objectively reflect the actual greenness of land cover. A remote-sensing image was obtained from Landsat8 (OLI_TIRS) on 23 July 2016, with a resolution of 30m * 30m. After processes of radiometric correction, atmospheric correction, etc., near-infrared bands and visible red bands are extracted. Then the value of NDVI is calculated. Accordingly, mean NDVI and green space quality within each sub-district can be obtained. Using Landsat to acquire NDVI data is one of the important applications, but because of the influence of aerosol optical thickness, certain bias may exist in term of the quality of the NDVI data [68,69]. 

#### 2.4.4. Point of Interest Data

Point of interest (POI) data is an important type of geospatial big data. Compared with remote sensing data and population density data, POI data features higher updating speed and lower acquisition cost [70]. Generally, POI data contains points with spatial features of name, category, longitude, latitude, etc. Spatial objects like building, residential area, park, school, hospital, company, shopping mall all could be point of interest. In this study, we acquired POI data from Baidu Maps and grouped them into four functional categories: Living, working, education and entertainment. Specifically, POIs subject to living includes residence, living service facilities (such as post offices, laundries, barber shops, and telecommunication business halls), shopping facilities (such as shopping malls, supermarkets, and convenience stores) and catering facilities (such as restaurants, snack bars, and fast food chains). POIs that fall under the category of working mainly consist of various enterprises and companies. Education POIs consist of all kinds of schools, such as kindergartens, high schools, universities, vocational schools, etc. KTV, bars, cinemas, recreation clubs, fitness centers, gyms, and some other similar facilities are treated as entertainment POIs.

### 2.5. Variable Calculation

#### 2.5.1. Dependent Variable

Public green space use of each sub-district is measured by the relative population density within it. The values of Wechat–Yichuxing are real-time data; therefore, the average relative population density of all the public green spaces in each sub-district is calculated to represent the PGS use. The detailed calculation formulas are as follows:

(1) $$P_{i}=\frac{\sum_{j=1}^{n}Q_{ij}}{d_{i}\sum_{j=1}^{n}G_{ij}}\;,$$

where $P_{i}$ is the relative population density of public green space in sub-district *i*, $d_{i}$ is the residential population density of sub-district *i* (this study takes it as a weight, when calculating the public green space use to reduce the influence of number difference of inhabitants in sub-district), $Q_{ij}$ is the average relative population within public green space *j* that belongs to sub-district *i*, $G_{ij}$ is the area of public green space *j* that belongs to sub-district *i* (in square meter), and *n* is the total numbers of public green space within sub-district *i*.

In particular,

(2) $$Q_{j}=\frac{\sum_{h=1}^{w}\sum_{t=1}^{m}X_{jt}}{wm}\;,$$

Where $Q_{j}$ is the average relative population of public green space j per day of the week (7 days), $X_{jt}$ is the relative population within the public green space *j* at time *t*, *m* is the total time points per day (here is 9), *w* is the total days. Here w is equal to 7. 

#### 2.5.2. Independent Variable

The connectivity of PGS is measured by the Integral Index of Connectivity in landscape ecology theory, which has been applied widely to study the connectivity of urban green space [38,71]. By referring to the relevant publications [33,59], the formula applied in our study is as below:

(3) $$IIC=\frac{\sum_{i=1}^{n}\sum_{j=1}^{n}\frac{a_{i}\cdot a_{j}}{1+nl_{ij}}}{A_{L}^{2}}\;,$$

where *IIC* is the integral index of connectivity; $a_{i}$ and $a_{j}$ respectively represent the areas of urban green space *i* and *j* (in square meters); $nl_{ij}$ means the link numbers between public green space *i* and *j* (based on the topological distance), and $A_{L}$ is the area of individual sub-district (in square meter). The value of *IIC* ranges from 0 to 1 and a higher value means higher connectivity.

With the Euclidean distances, we calculate the *IIC* by Conefor 2.6 [72]. Considering the human walking speed and the acceptable walking distance [5,10,73,74], only the PGSs within 500 m are considered for *IIC* calculation.

#### 2.5.3. Relevant Impact Factors

The greenness of each study unit is measured by the mean NDVI, which is derived by zonal analysis in ArcGIS 10.2 (ESRI, Redlands, CA, USA), based on the processed remote-sensing image. Average PGS area per capita (with the unit of ha/per capita) is obtained from the calculation of the total PGS area (consisting of all PGS, not merely the ones considered above) being divided by the number of residents. The ratio of arbor to shrub can mirror the quality of green space to a certain degree, and when the value reaches about 1:1.5, it represents a high-quality landscape [22]. Therefore, the ratio of arbor to shrub is applied to indicate the quality of PGS in this study. The data of arbor and shrub are extracted from NDVI by referring to the suggested classification standard [75], and local context in Wuhan. As shown in Table 1, the function of each analysis unit is defined by the density of various POIs.

### 2.6. Analysis Method

Before analysis, data centering (mean-subtraction) is first applied. Then, the direct relationship between the connectivity of public green space and its use is explored. Following this is the analysis of the moderation/mediation effect of the selected impact factors on such relationship. All through this process, regression analysis is applied by using SPSS 20 (IBM Corporation, Armonk, NY, USA) and the plugin of Process version 3.4 (Andrew F. Hayes, Ohio State University, USA) (Downloaded from http://processmacro.org/download.html).

**Figure 3:**
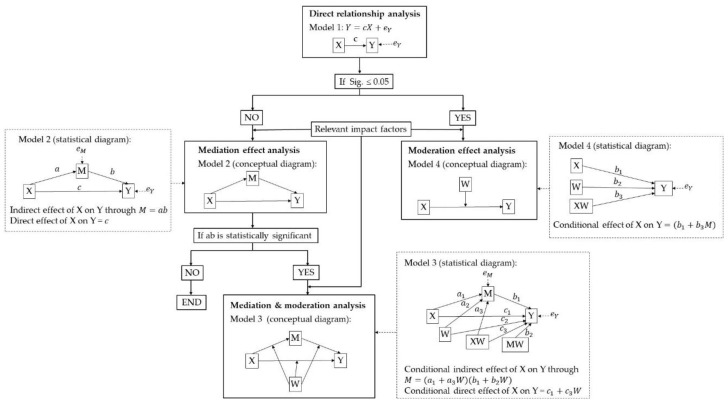
Analysis method.

As described in Figure 3, we deploy Model 1 first to analyze the correlation between the independent variable X (IIC) and the dependent variable Y (PGS use) without consideration of any other factors. If the correlation between IIC and PGS use exists, then we will apply Model 4 and directly take those related factors as the moderators to analyze their influence on this relevance. However, if there is no statistically significant relevance between them, then we will suppose that some impact factors might mediate the relevance between them. That is to say, connectivity (IIC) may need the mediation of other impact factors to exert influence upon PGS use. Using Model 2, we can test this guess. If the indirection effects of IIC on PGS use, namely ab in Figure 2, are statistically significant, this means that the supposition at this step is acceptable and IIC cannot affect PGS use independently. In this circumstance, we then need to proceed to examine how PGS use is influenced by IIC with the mediation of these identified factors, and whether the rest of the selected factors have an impact through this pathway (Model 3). 

## 3. Results

**Figure 4:**
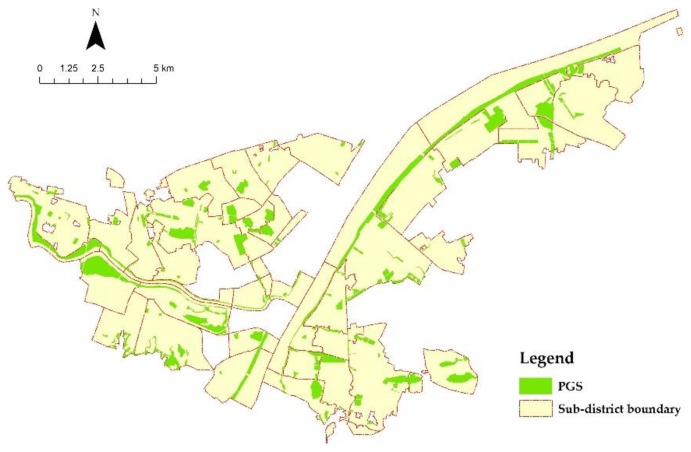
Distribution of PGS (with an area of more than 1ha).

**Figure 5:**
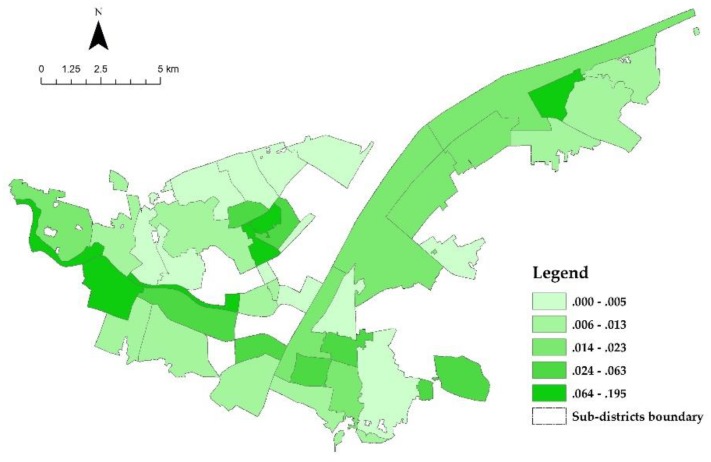
Distribution of IIC.

**Figure 6:**
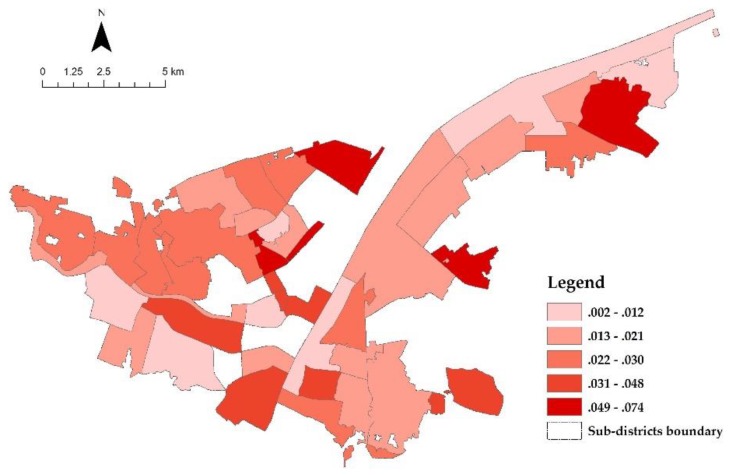
Distribution of PGS use.

The selected PGS distribution is depicted in Figure 4. Except for the greenbelt alongside the Yangtze River and Han River, most of the PGSs are sporadically dispersed within the unit of the sub-district. When mapping the calculation results of IIC, it is not hard to find that the IIC distribution is correspondent with the configuration of PGS (shown in Figure 5). Different from IIC distribution, the distribution of PGS use seems to be more random (Figure 6). Some sub-districts with higher IIC have higher PGS use, while some others have a contrary landscape. Nevertheless, by comparing it with the results of accessibility analysis by Xing et al. (2018) [55], we find that most of those sub-districts with higher PGS use have relatively good accessibility to PGS. It is a manifestation of the significance of accessibility in PGS use.

The descriptive characteristics of those selected indicators are illustrated in Table 2. The maximum of IIC is 0.195, which suggests that the connectivity of PGS in the Wuhan old town area is at a relatively low level. The average PGS use (unit: capita/ha) is low, and some PGSs are even seldom used, which might be due to the low accessibility of PGS. Furthermore, the areas with high accessibility are dispersedly distributed in terms of walking distance. Most of the residents need to walk 30-35 min to arrive at PGSs. As a result, they are not willing to spend that much time walking to a PGS and do not use it. Although the greenness of several sub-districts is relatively high, the NDVI values of some sub-districts (consisting parts of the Yangtze River and Han River) are negative. It is worth noting that a low NDVI value does not necessarily mean a bad natural environment in this study, as it might be as a result of the blue waters. For this, the ratio of arbor to shrub—an indicator to measure the green space quality—should be accounted for in the real PGS situation. According to the table below, the ratios of arbor to the shrub in most sub-districts are lower than the suggested value of 0.667 (with more shade effect), but half of them are higher than 0.345 (with a greater landscape effect) [65]. The difference between the average PGS area per capita between maximum and minimum is significant. Compared with 10.91 m^2^ per capita of PGS area at the whole- city level [76], PGS area per capita in our study scope is obviously lower. From the perspective of the land-use POI density, the main land-use types are for catering facilities and companies, and the land-uses for education is the lowest within the study area. That is to say, compared with education and entertainment, land-uses functioning for living and working dominate in those selected sub-districts.

In Model 1, the Significance (Sig.) value of the regression analysis is 0.397, which is much greater than 0.05. It means that Model 1 has no statistical significance. Therefore, we can infer that the weak correlation between IIC and PGS use (R=0.134) without consideration of other impact factors is invalid in a statistical sense (Table 3). Therefore, we can say that IIC (ranging from 0 to 0.195) cannot directly influence PGS use in this study area. By following the analysis methods (Figure 3), we input each selected impact factor into Model 2 to test if it will act as the mediator to make the regression model significant.

As has been explained, if the direct effect of IIC on PGS use is statistically significant, then this means that IIC can affect PGS use directly; and if it is not, then this implies that IIC may affect PGS use through other mediators [77]. Since we have proved that IIC does not influence PGS use directly in Model 1, we then must check the significance of the indirect effect from the mediators in Model 2.

The details of Model 2 are listed in Table 4. The confidence interval of the indirect effect ranges from a negative value to a positive value and includes zero, which means that the each of the listed factors has no indirect effects upon PGS use in a statistical sense [77]. In other words, there is no impact factor identified as being significant at this step and the assumption of Model 2 should be rejected. The selected impact factors that have the potential to influence PGS use cannot mediate the relationship between IIC and PGS use in this study. The results of the direct effect of X on Y illustrated in Table 4 are consistent with what Model 1 has revealed, which proves again that IIC cannot directly affect PGS use. 

Intriguingly, when treating the density of company as the mediator and inputting it into Model 2, we find that it positively correlates with PGS use (Table 5). This outcome implies that land−use for companies have the potential to promote PGS use. In contrast, all the other impact factors are proved to have no similar relationship with PGS use.

## 4. Discussion

According to the analysis results above, IIC has no direct influence on PGS use, even if we take potentially relevant impact factors into consideration. It might be a result of the overall very low connectivity of PGS in Wuhan old town. The maximum IIC is 0.195 in this study, which is far lower than 1. In other words, it is possible that a slight change in the connectivity of PGS will have very a limited effect upon the change of PGS use. This may be similar with other green space indicators. For example, PGS area per capita will generate the effect of promoting green space use, only if it reaches a certain threshold [63]. By following this logic, we may say that only if IIC increases by a certain higher degree will its improvement lead to the significant promotion of PGS use. 

Moreover, a high connectivity of green space can represent good accessibility between green spaces to a certain degree; however, this does not also imply that green spaces can be accessed easily from other non−green places (such as the residences, schools, and workplaces), which needs people access to the green space system first if they want to use it. According to previous studies on green space in Wuhan, the old town areas have very low PGS accessibility [54,55,78]. Therefore, the ignorable weak relationship between the connectivity of PGS and PGS use might be partly attributed to the poor initial accessibility of PGSs. In addition, once a person gets into a PGS, whether they will stay in this one or move to another PGS is influenced by a variety of factors, such as the recreational demands and the facilities in green space, as well as if they are in a group [61]. Connectivity among PGS may not be powerful enough to encourage PGS users to move to other PGSs. Therefore, we may conclude that, without the precondition of better accessibility and decisive attractors, improvement of connectivity may have a very limited impact upon PGS use.

According to the existing literature, indicators of NDVI, PGS area per capita, PGS quality, and the facilities for residence, commercial, etc. are correlated to green space use [18,41,60,63]. In this study, however, this relationship is not evident. This might be due to the difference in the selection standard of PGS between this study and the others. In this research, only the PGSs larger than 1ha were used; thus, small ones were neglected. However, Cohen et al. (2014) found that pocket parks are usually used by residents at the same frequency or even at a higher frequency than the neighborhood parks [79]. That is to say, the neglection of the pocket parks might result in the difference in the conclusion. In addition, Tilt et al. (2007) also did not find a significant relationship between NDVI and green space use [80], but they reported that the perception of greenness is related to the improvement of physical activity, while NDVI has an impact on people’s perception of accessibility to a certain destination. According to this point, we can infer that the relatively low NDVI in Wuhan old town might worsen the perception of accessibility that is already poor in reality, and subsequently influence PGS use. Park et al. (2013) reported that PGS area per capita could promote moderate and vigorous intensities of physical activity, while there is no contribution to low− intensity physical activity [63]. However, in Wuhan old town, PGSs are more likely to be used for partaking in low−intensity of physical activity [81]. That might influence the correlation between PGS area per capita and PGS use in this study. In addition, the ratio of arbor to shrub to represent PGS quality may not represent the full landscape of green space quality. In fact, other indicators, such as available amenities, maintenance, and safety are also keys to PGS quality, and they have been proven to have the potential to improve PGS use.

The study has also found that land−use types surrounding PGS have roughly no significant correlation with PGS use. On the one hand, the PGSs that are located in highly residential and commercial areas in Wuhan old town are small [41]. As has been explained, the area of PGS considered in this study is larger than 1ha, and thus, those relatively small ones are neglected. Therefore, real PGS use may be higher than what has been calculated. On the other hand, a variety of facilities might improve PGS use, while the poor characteristics of PGS, such as low PGS area per capita and NDVI, have adverse impacts on it. Therefore, these two opposite influences will result in a trade−off between improvement and deterioration in terms of PGS use. 

We found that the density of companies has a positive relationship with PGS use, even though it is not dominantly strong. As mentioned above, the measurement of PGS use is based on the data of Yichuxing, which is obtained from WeChat users. According to the Wechat Data Report in 2018, more than 90% of them are younger than 55 years old [82]. Therefore, the users who were analyzed to represent the PGS use are more likely employed, and they spend most of their walking time moving around their workplace. However, the elderly residents, particularly in the old town areas, are more likely to use PGSs that are close to their residence. This indirectly explains why the density of companies can promote PGS use, but not the density of residences. Moreover, the positive relationship between the density of companies and PGS use aligns with the growing body of empirical research that workplace green space contributes to reducing the work−related stress and improving employee satisfaction, as well as their well−being [83,84,85]. In turn, companies may encourage employees to use green space around the workplace [86].

Before drawing the conclusions, a few points need to be stressed here. First, the non−correlation between IIC and PGS use might just exist in a certain range of PGS connectivity. In other words, the independent variable of IIC (the connectivity of PGS) in the whole study area is relatively low (ranging from 0 to 0.195) and the value of each unit (sub−district) does not vary significantly in this study. That is to say, we should confine our conclusion to the connectivity value range of (0, 0.195). In the situation where PGS connectivity is higher than 0.19, if this conclusion can still stand is unknown. Therefore, extending the range of the independent variable value by changing the study unit is necessary for further research. Moreover, the connectivity of PGS works in promoting its use by human beings is based on the precondition of good availability and accessibility of PGS. However, the poor characteristics of PGS in the Wuhan old town area, on the one hand, decreases local residents’ access to PGS from other non−green space, and on the other hand, this affects the movement of PGS users from one PGS to another. 

More importantly, there are still several issues that need further exploration. First, although LBS data has the merit of fine spatiotemporal granularity, such as real−time and relatively precise geo−location, we must admit that the indicator of PGS use based on LBS data is supplementary to traditional data obtained by means of a questionnaire, observation, etc., but not a substitute. Big−data—like Yichuxing applied in this study—have inherent limitations. For example, the lack of demographic and socioeconomic information of PGS users make it impossible to analyze how an individual’s socioeconomic status would influence PGS use. Moreover, users of WeChat tend to be younger than 55 years old. It means that most people older than 55 are not counted in this study, which might cause a biased conclusion. Hence, collecting individual information by the survey is necessary for a deeper understanding of PGS use. Besides, as some quality−related data cannot be obtained without investigating them one by one, indicators of these aspects that have the potential to influence PGS use are not included. Instead, we use the ratio between arbor and shrub that can be derived from remote sensing image. This manipulation might cause an inaccurate representation of the real situation of the green space quality. Moreover, in order to keep the spatial analysis unit consistent with the obtained official data of census (the unit of sub−district level), we apply the vector map of sub−district distribution in 2014. However, as Wuhan is still at a stage of massive construction in recent years. As a result, the administrative boundary of several sub−districts has been adjusted over the past five years, which means the calculation results of PGS use and IIC could be different in different years. To avoid this issue, one possible solution is to use the product of the average population per residential land in the district level and the total residential land in the sub−district level to represent the population of a certain sub−level district. Last, but not the least, considering the rapid change of the road system (as a result of massive ongoing construction) in Wuhan, we use Euclidean distance instead of the road routine distance to represent the distances among PGSs as it would not change over time despite the change of the road system. To calculate IIC, a Euclidean distance of 500 m has been applied. However, it should be admitted that Euclidean distance-based IIC, to some extent, might not accurately reflect the real connectivity of PGSs. In addition, the application of the distance of 500 m might exclude those who have used certain PGS, but live more than 500 m away from the PGS. As a result, it could potentially influence the correlation between connectivity and PGS use. Therefore, in the condition that road change is not obvious, and official road network data are available in the future, it would be more reasonable to use road routine distance for IIC calculation and regression analysis. 

## 5. Conclusions

Instead of focusing on the ecology−related influence of connectivity, we explore the health−related influence (represented by PGS use) of connectivity on human beings by using LBS data. This is a pioneer effort to analyze the relationship between PGS connectivity and PGS use by human beings using LBS data of Yichuxing in Wuhan. In order to explore if improvement of the connectivity of PGS contributes to PGS use, we applied Regression Analysis to test the relationship between them. The analysis results are different from what we expected in a general sense. No statistically significant correlation is found between the connectivity of PGS and PGS use. For one thing, that might be due to the modifiable areal unit problem, which results in the very small value range (0, 0.19) of the connectivity of PGS. Thus, the above conclusion should not be extended to other conditions with connectivity of PGS higher than 0.19. For another, the relationship between the connectivity of PGS use is affected by many factors, especially the characteristics of PGS (including accessibility, availability, quality, etc.). As the accessibility of PGS in the Wuhan old town area is low, the residents’ intentions and actions to use the PGS are limited. Therefore, the residents’ access to a certain PGS (related to accessibility) as the first step, and movement among PGSs (related to connectivity) as the second step cannot be well built. Thus, focusing on connectivity improvement, but no betterment of accessibility may be in vain in terms of increasing PGS use. Nevertheless, we find that the density of companies is positively related to PGS use. This finding is consistent with the conclusions drawn by other studies that the PGSs near workplaces benefits employees both physically and psychologically, and there is an increasing trend that workers are likely to use the PGS surrounding their workplace.

## Figures and Tables

**Table 1:** The variables and the requisite data.

Category	Variables	Description	Data Source		Type
			Special Data	Basic Data	
Independent variable	the connectivity of PGS in each sub-district	measured by IIC	GS distribution	sub-district distribution	numeric
Dependent variable	PGS use	measured by relative population density of PGS within each sub-district	Wechat-Yichuxing; GS distribution; residents within each sub-district		
Impact factors	**Green space characteristics**				
	the greenness of each sub-district	measured by mean NDVI	remote sensing image from Landsat8 (OLI_TIRS) on 23 July 2016 with 30m * 30 m		
	the quality of PGS	measured by RAS			
	the availability of PGS	measured by PGSAPC	GS distribution; residents within each sub-district		
	**Facility function characteristics**				
	Facilities for living	measured by the density of residence	residence POI		
		measured by the density of LSF	LSF POI		
		measured by the density of CF	CF POI		
		measured by the density of SF	SF POI		
	Facilities for working	measured by the density of company	company POI		
	Facilities for education	measured by the density of school	school POI		
	Facilities for entertainment	measured by the density of SLF	SLF POI		

Note: Integral Index of Connectivity = IIC; Public Green Space = PGS; Green Space = GS; Normalized Difference Vegetation Index = NDVI; Ratio of Arbor to Shrub = RAS; Public Green Space Area Per Capita = PGSAPC; Point of Interest = POI; Living Service Facilities = LSF; Catering Facilities = CF; Shopping Facilities = SF; Sport and Leisure Facilities = SLF.

**Table 2:** Descriptive statistics (before mean-subtraction).

Variables	N	Range	Minimum	Maximum	Mean		Std. Deviation	Variance
	Statistic	Statistic	Statistic	Statistic	Statistic	Std. Error	Statistic	Statistic
IIC	42	0.194	0.000	0.195	0.029	0.007	0.042	0.002
PGS use	42	0.071	0.002	0.074	0.028	0.003	0.018	0.000
NDVI	42	0.780	−0.213	0.567	0.267	0.026	0.166	0.028
RAS	42	1.201	0.000	1.201	0.363	0.037	0.238	0.057
PGSAPC	42	34.640	0.210	34.850	7.273	1.244	8.062	64.993
density of CF	42	5.538	0.118	5.656	1.276	0.152	0.985	0.970
density of LSF	42	1.931	0.039	1.970	0.501	0.058	0.374	0.140
density of residence	42	1.033	0.020	1.053	0.320	0.035	0.228	0.052
density of SF	42	2.155	0.066	2.221	0.668	0.068	0.440	0.193
density of company	42	5.607	0.106	5.713	1.067	0.167	1.081	1.169
density of school	42	0.576	0.008	0.583	0.103	0.016	0.102	0.010
density of SLF	42	0.972	0.013	0.985	0.330	0.033	0.214	0.046
Valid N (listwise)	42							

Note: Integral Index of Connectivity = IIC; Public Green Space = PGS; Normalized Difference Vegetation Index = NDVI; Ratio of Arbor to Shrub abbr RAS; Public Green Space Area Per Capita = PGSAPC; Living Service Facilities = LSF; Catering Facilities = CF; Shopping Facilities = SF; Sport and Leisure Facilities = SLF.

**Table 3:** Detailed analysis results of Model 1.

Model Summary					
R	R Square	Adjusted R Square	Std. Error of the Estimate	Durbin–Watson	Variables
0.134	0.018	−0.007	0.01827	2.027	Y: PGS use; X: IIC
**ANOVA**					
	Sum of Squares	df	Mean Square	F	Sig.
Regression	0.000	1	0.000	0.734	0.397
Residual	0.013	40	0.000		
Total	0.014	41			
**Coefficients**					
	Unstandardized Coefficients		Standardized Coefficients	t	Sig.
	B	Std. Error	Beta		
(Constant)	0.000	0.003		0.000	1.000
IIC	−0.058	0.068	−0.134	−0.857	0.397

Note: X represents independent variable; Y represents dependent variable.

**Table 4:** The details of Model 2 about the direct and indirect effect of IIC on PGS use.

Variables		Direct effect of X on Y				Indirect effect(s) of X on Y		
X and Y	M	Effect	p	LLCI	ULCI	Effect	BootLLCI	BootULCI
X: IIC; Y: PGS use	PGSAPC	−0.026	0.733	−0.176	0.125	−0.032	−0.100	0.105
	RAS	−0.058	0.405	−0.197	0.081	0.000	−0.036	0.029
	NDVI	−0.055	0.422	−0.191	0.082	−0.003	−0.043	0.021
	density of CF	−0.061	0.365	−0.196	0.074	0.003	−0.026	0.043
	density of LSF	−0.071	0.295	−0.205	0.064	0.013	−0.031	0.059
	density of residence	−0.067	0.333	−0.205	0.071	0.009	−0.026	0.035
	density of school	−0.060	0.379	−0.198	0.077	0.003	−0.014	0.027
	density of SF	−0.059	0.387	−0.195	0.077	0.001	−0.033	0.024
	density of SLF	−0.089	0.212	−0.230	0.053	0.031	−0.030	0.101
	density of company	−0.121	0.070	−0.252	0.011	0.063	−0.046	0.206

Note: (1) X represents independent variable; Y represents dependent variable; M represents mediators. (2) Integral Index of Connectivity = IIC; Public Green Space = PGS; Normalized Difference Vegetation Index = NDVI; Ratio of Arbor to Shrub = RAS; Public Green Space Area Per Capita = PGSAPC; Living Service Facilities = LSF; Catering Facilities = CF; Shopping Facilities = SF; Sport and Leisure Facilities = SLF. (3) Level of confidence for all confidence intervals in the output: 95.0000. (4) Number of bootstrap samples for percentile bootstrap confidence intervals: 5000.

**Table 5:** Detailed analysis results of Model 2 (mediator: Density of company).

OUTCOME VARIABLE: PGS Use						
Model Summary						
R	R−sq	MSE	F	df1	df2	*p*
0.4536	0.2058	0.0003	5.0528	2	39	0.0112 *
Model						
	coeff	se	t	p	LLCI	ULCI
constant	0	0.0026	0	1	−0.0052	0.0052
IIC	−0.121	0.065	−1.8617	0.0702	−0.2524	0.0105
density of company	0.0077	0.0025	3.0365	0.0043 **	0.0026	0.0128

Note: (1) Level of confidence for all confidence intervals in the output: 95.0000. (2) ** means *p* value is no more than 0.01; * means *p* value is no more than 0.05.
